# Supplementary material for: Panorama: A robust pangenome-based method for predicting and comparing biological systems across species
Source: PLoS Comput Biol. 2026 Jul 10;22(7):e1013856. doi: 10.1371/journal.pcbi.1013856 (PMC13379101; doi:10.1371/journal.pcbi.1013856)

**S1 Fig. Computational performance of the analysis pipeline across bacterial species.** Stacked bar chart showing the execution time (in minutes) for each step of the workflow across species with varying numbers of genomes (black line, right y-axis). The four pipeline steps are: Load (blue) - time to load the pangenome data; Annotation (green) - time to annotate gene families with defense system function with HMMs; Detection (yellow) - time to detect defense systems; and Projection (red) - time to project defense systems across genomes. The benchmark was conducted on a Linux server with two Intel Xeon Gold 6150 processors (36 cores), 376 GiB RAM, running CentOS v7.9.2009 with Python 3.10. Execution time scales with dataset size, with the projection step exhibiting super-linear scaling.

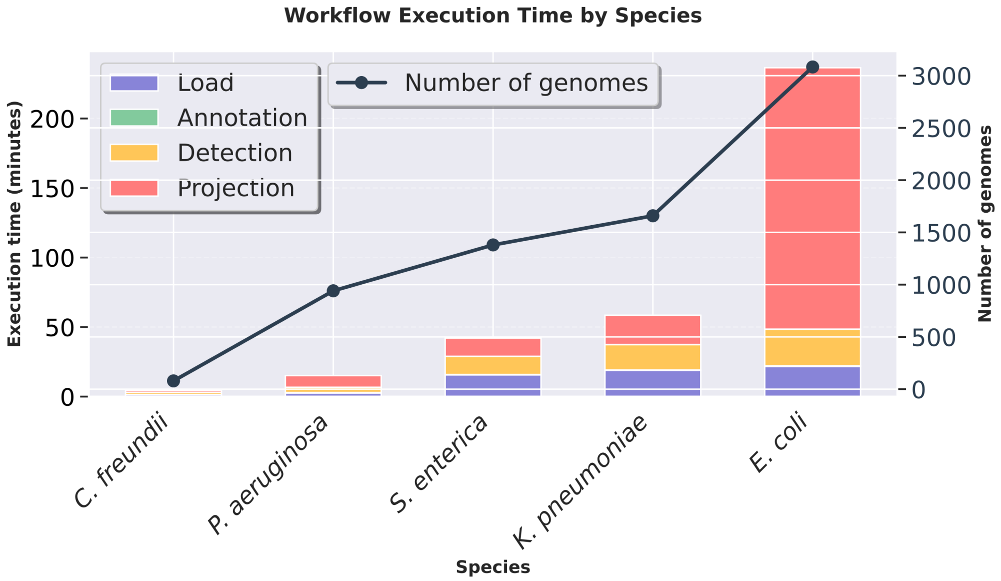

Supplement: S1 Fig — Stacked bar chart showing the execution time (in minutes) for each step of the workflow across species with varying numbers of genomes (black line, right y-axis). The four pipeline steps are: Load (blue) - time to load the pangenome data; Annotation (green) - time to annotate gene families with defense system function with HMMs; Detection (yellow) - time to detect defense systems; and Projection (red) - time to project defense systems across genomes. The benchmark was conducted on a Linux server with two Intel Xeon Gold 6150 processors (36 cores), 376 GiB RAM, running CentOS v7.9.2009 with Python 3.10. Execution time scales with dataset size, with the projection step exhibiting super-linear scaling. (PDF) [file pcbi.1013856.s001.pdf]
