# Supplementary material for: Panorama: A robust pangenome-based method for predicting and comparing biological systems across species
Source: PLoS Comput Biol. 2026 Jul 10;22(7):e1013856. doi: 10.1371/journal.pcbi.1013856 (PMC13379101; doi:10.1371/journal.pcbi.1013856)
Supplement: S1 Table — Execution time (in minutes:seconds format) for each step of the analysis workflow across five bacterial species with varying dataset sizes. PPanGGOLiN: time to construct, partition and analyse (RGPs, Spots and modules) the pangenome graph; Load: time to load the pangenome data; Annotation: time to annotate gene families with defense system functions from HMMs; Detection: time to detect defense systems; Projection: time to project defense systems across genomes. Analyses were performed on two Intel Xeon Gold 6150 processors (36 cores), 376 GiB RAM, with Python 3.10. Execution time scales with the number of genomes, with the projection step showing the most substantial increase in larger datasets. P. aeruginosa exception can be explained by the use of fasta file in the pangenome construction, adding an annotation step in PPanGGOLiN workflow. (PDF) [file pcbi.1013856.s002.pdf]

S1 Table. Execution time of the workflow across multiple species. Execution time (in minutes:seconds format) for each step of the analysis workflow across five bacterial species with varying dataset sizes. PPanGGOLiN: time to construct, partition and analyse (RGPs, Spots and modules) the pangenome graph; Load: time to load the pangenome data; Annotation: time to annotate gene families with defense system functions from HMMs; Detection: time to detect defense systems; Projection: time to project defense systems across genomes. Analyses were performed on two Intel Xeon Gold 6150 processors (36 cores), 376 GiB RAM, with Python 3.10. Execution time scales with the number of genomes, with the projection step showing the most substantial increase in larger datasets. *P. aeruginosa* exception can be explained by the use of fasta file in the pangenome construction, adding an annotation step in PPanGGOLiN workflow.

Species	Number of genomes	PPanGGOLiN	Load	Annotation	Detection	Projection
C. freundii	79	01:18.98	01:22.18	00:09.07	01:32.47	01:17.98
P. aeruginosa	941	36:17.30	02:33.70	00:20.60	03:47.50	08:22.23
S. enterica	1380	22:07.31	15:38.76	00:12.10	13:06.14	13:09.22
K. pneumoniae	1659	34:10.82	18:51.78	00:17.69	18:15.45	21:04.96
E. coli	3083	58:34.13	21:36.71	00:26.32	26:26.29	188:02.75
