# Supplementary material for: Panorama: A robust pangenome-based method for predicting and comparing biological systems across species
Source: PLoS Comput Biol. 2026 Jul 10;22(7):e1013856. doi: 10.1371/journal.pcbi.1013856 (PMC13379101; doi:10.1371/journal.pcbi.1013856)

S3 Fig. Species-specificity evaluation across system categories in Enterobacteriaceae pangenomes. The enrichment factors were computed using the method described in *enrichment factor equation*, providing a quantitative measure of species-specific representation.

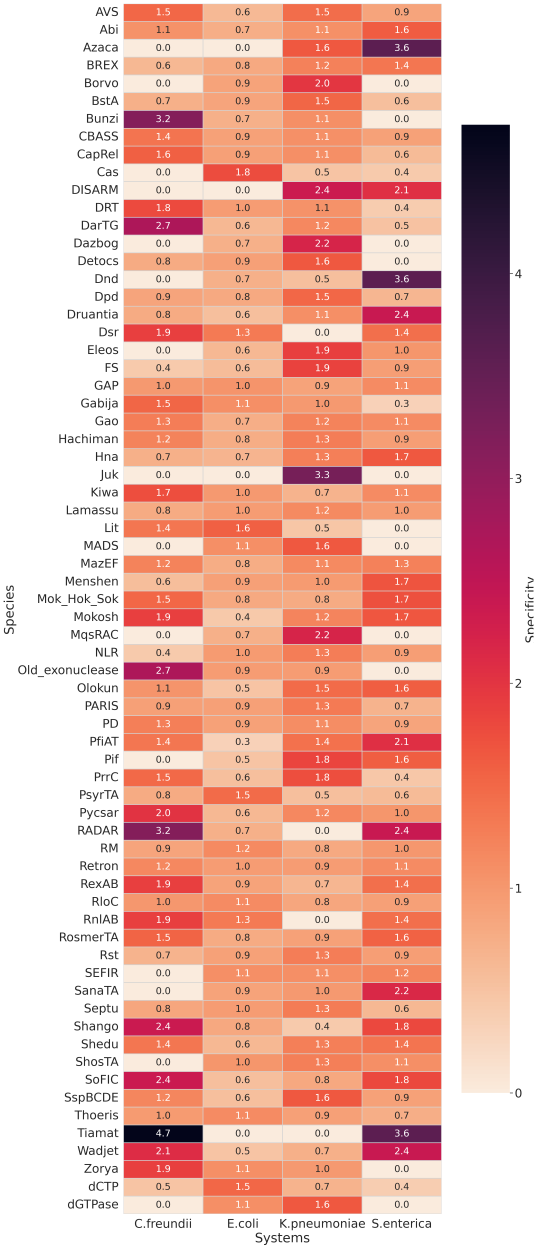

Supplement: S3 Fig — The enrichment factors were computed using the method described in Eq 6, providing a quantitative measure of species-specific representation. (PDF) [file pcbi.1013856.s004.pdf]
