# Supplementary material for: Panorama: A robust pangenome-based method for predicting and comparing biological systems across species
Source: PLoS Comput Biol. 2026 Jul 10;22(7):e1013856. doi: 10.1371/journal.pcbi.1013856 (PMC13379101; doi:10.1371/journal.pcbi.1013856)

S4 Fig. Defense systems within insertion spots. The bar plot displays the number of predicted defense systems in the pangenome for each spot of insertion. (A) *E. coli* pangenome. (B) *S. enterica* pangenome.

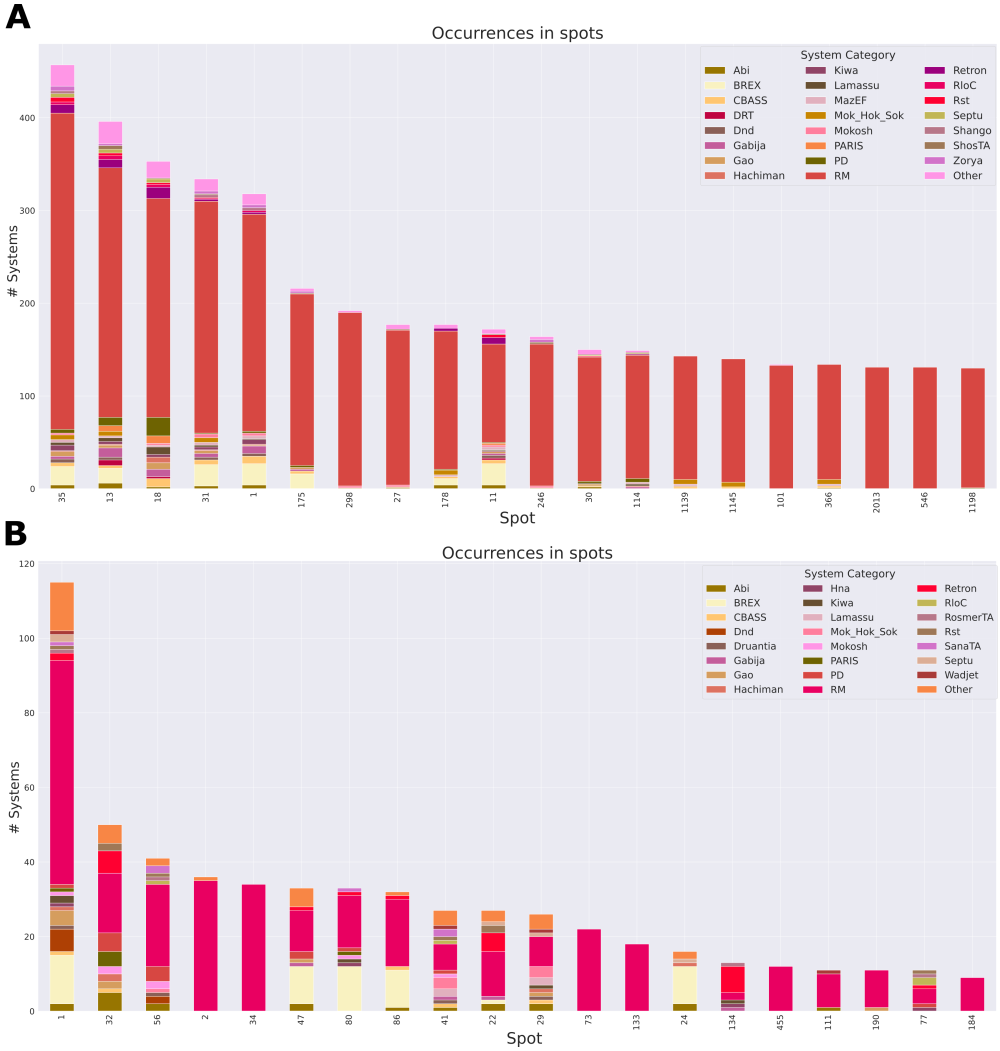

Supplement: S4 Fig — The bar plot displays the number of predicted defense systems in the pangenome for each spot of insertion. (A) E. coli pangenome. (B) S. enterica pangenome. (PDF) [file pcbi.1013856.s005.pdf]
