# Supplementary material for: Panorama: A robust pangenome-based method for predicting and comparing biological systems across species
Source: PLoS Comput Biol. 2026 Jul 10;22(7):e1013856. doi: 10.1371/journal.pcbi.1013856 (PMC13379101; doi:10.1371/journal.pcbi.1013856)

S5 Fig. Defense system composition of spots belonging to cluster 3062. Distribution of defense systems in two representative spots from cluster 3062: spot 175 in *E. coli* (A) and spot 86 in *S. enterica* (B). Each slice represents the proportion of a given defense system category within the spot. RM (Restriction-Modification) systems dominate both spots, accounting for 81.3% in *E. coli* and 57.1% in *S. enterica*. BREX represents the second most abundant system in *S. enterica* (23.8%), while it is present at lower frequency in *E. coli* (6.6%). Other defense systems are present at lower frequencies.

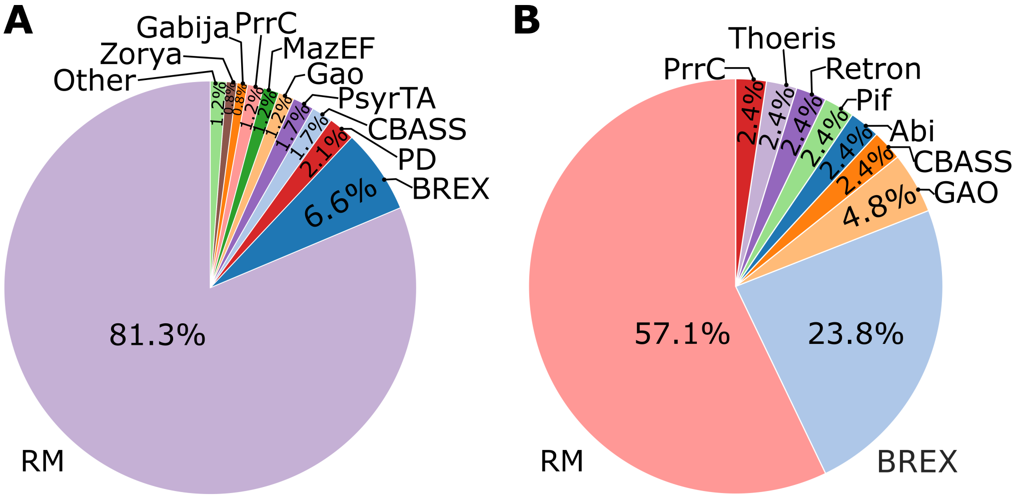

Supplement: S5 Fig — Distribution of defense systems in two representative spots from cluster 3062: spot 175 in E. coli (A) and spot 86 in S. enterica (B). Each slice represents the proportion of a given defense system category within the spot. RM (Restriction-Modification) systems dominate both spots, accounting for 81.3% in E. coli and 57.1% in S. enterica. BREX represents the second most abundant system in S. enterica (23.8%), while it is present at lower frequency in E. coli (6.6%). Other defense systems are present at lower frequencies. (PDF) [file pcbi.1013856.s006.pdf]
