# Supplementary material for: Panorama: A robust pangenome-based method for predicting and comparing biological systems across species
Source: PLoS Comput Biol. 2026 Jul 10;22(7):e1013856. doi: 10.1371/journal.pcbi.1013856 (PMC13379101; doi:10.1371/journal.pcbi.1013856)

S2 Fig. Example of PANORAMA automated output: number of systems predicted per model across Enterobacteriaceae pangenomes. This heatmap is automatically generated by PANORAMA using the Bokeh package [1]. Each row corresponds to a species pangenome and each column to a defense system model; color intensity indicates the number of predicted systems (color scale, right). The figure is shown here to illustrate the format of PANORAMA's built-in visualization output. The full interactive version — enabling zooming, panning, and hovering to display exact values — is available in the Zenodo repository and can be opened directly in any standard web browser.

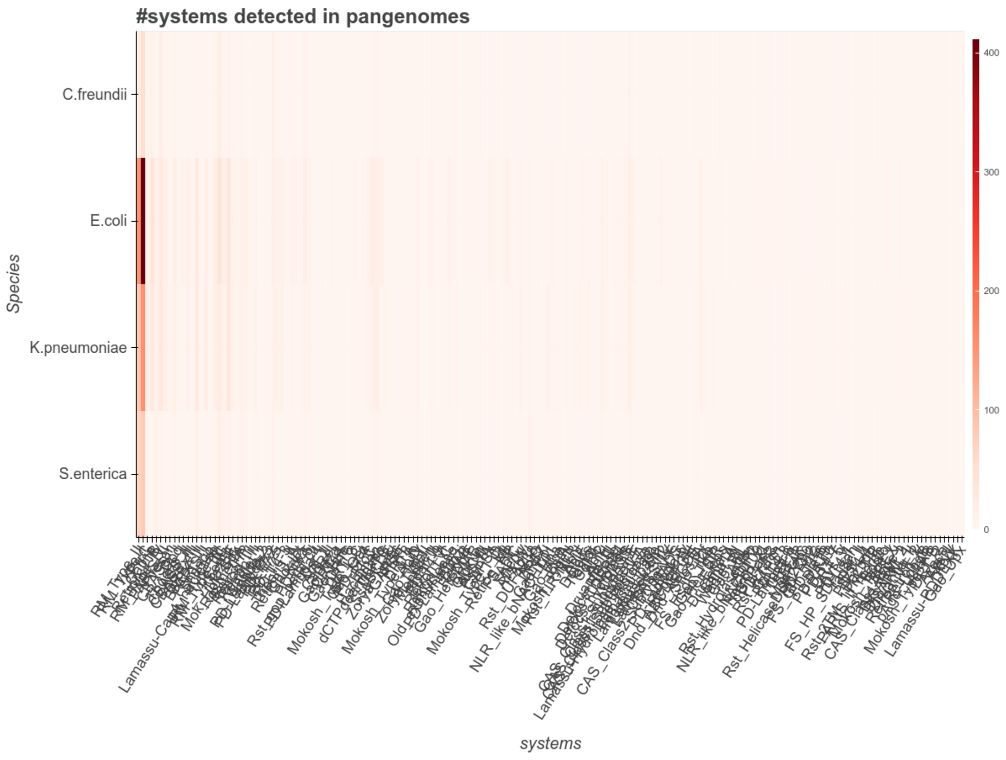

Supplement: S2 Fig — This heatmap is automatically generated by PANORAMA using the Bokeh package [53]. Each row corresponds to a species pangenome and each column to a defense system model; color intensity indicates the number of predicted systems (color scale, right). The figure is shown here to illustrate the format of PANORAMA’s built-in visualization output. The full interactive version — enabling zooming, panning, and hovering to display exact values — is available in the Zenodo repository and can be opened directly in any standard web browser. (PDF) [file pcbi.1013856.s003.pdf]
