# Supplementary material for: Panorama: A robust pangenome-based method for predicting and comparing biological systems across species
Source: PLoS Comput Biol. 2026 Jul 10;22(7):e1013856. doi: 10.1371/journal.pcbi.1013856 (PMC13379101; doi:10.1371/journal.pcbi.1013856)
Supplement: Attachment — Submitted filename: Response2Reviewers.pdf [file pcbi.1013856.s007.pdf]

Response to Reviewers

We thank the reviewers for their careful reading of the manuscript and their constructive comments. We have addressed each point below. Reviewer comments are shown in *italics*, followed by our responses. Changes to the manuscript are described and, where relevant, the revised text is provided.

Reviewer 1

Comment 1 — Discussion on model creation

Discussion of how to create models would be useful. It is not clear how much of this is implemented within PANORAMA itself.

Response:
We thank the reviewer for this comment. Model creation is fully implemented within PANORAMA itself, and we have added a dedicated **"Model creation and distribution"** section to the Materials and Methods to make this explicit.

PANORAMA provides 2 complementary pathways for model creation:

1. **Translation from existing tools**: Automatic conversion utilities translate models from MacSyFinder, DefenseFinder, CasFinder, and PADLOC into the PANORAMA JSON format, together with the associated HMM database input files. Since these tools are the primary source of models used with PANORAMA, this allows users to immediately leverage existing, validated model collections without any manual reformatting.
2. **Creating new models from scratch**: For novel systems not covered by existing databases, dedicated utilities generate both the model input files and the corresponding HMM database input files from user-supplied data.

Also, before any analysis is run, PANORAMA automatically checks the grammar and internal consistency of all model files, reporting errors and warnings so that issues can be corrected prior to execution.

A curated collection of ready-to-use models and HMM libraries is publicly available on the PANORAMA models GitHub organization (https://github.com/PANORAMA-models), allowing direct download and use without additional configuration. Full step-by-step documentation covering all three model creation pathways, including annotated examples, is available in the online documentation at https://panorama.readthedocs.io/latest/modeler/modeler_guide.html

We have also added a forward reference to this new subsection in the Overview paragraph of the Results section, and clarified there that model creation is handled directly within PANORAMA:

> *Models are stored in JavaScript Object Notation (JSON) format, with a flexible and easy-to-understand grammar, enabling users to customize or create new models. The complete creation workflow and available model collections are described in the Materials and Methods section.*

Comment 2 — Relevant tools to include

Other tools which may be relevant to cite include:
- Hamburger: https://www.nature.com/articles/s41467-022-32929-2
https://github.com/djw533/hamburger
- spacedust: https://www.nature.com/articles/s41592-025-02816-x
https://github.com/soedinglab/spacedust

Response:

We thank the reviewer for pointing out these two relevant tools. Hamburger and Spacedust address the identification of genomically co-localized gene clusters by scanning genomic neighborhoods in individual assemblies, without exploiting the graph-based representation of gene presence, absence, and chromosomal contiguity provided by a pangenome. In contrast, PANORAMA directly queries a gene adjacency graph to detect multi-gene systems at pangenome scale, capturing genomic context across all strains simultaneously. Furthermore, PANORAMA integrates system-specific detection models that define, for each target system, an expected combination of mandatory, accessory, neutral, and forbidden gene families — providing a level of biological specificity and interpretability that rule-agnostic neighborhood-scanning approaches cannot offer. Both tools have been cited in the introduction as related methods for gene cluster identification.

> *Several tools have been developed to detect macromolecular systems at the genome scale. For example, Hamburger identifies spatially constrained subsets of proteins within a defined genomic neighborhood and has been applied to the detection of Type VI secretion systems. As for Spacedust, it follows a different strategy, relying on protein structure comparisons and ordered conservation matrices; it has been applied, for example, to the detection of defense systems.*

Comment 3 — Gene co-occurrence detection methods

It may be relevant to note gene-gene co-occurrence detection methods such as Coinfinder or Goldfinder

https://www.microbiologyresearch.org/content/journal/mgen/10.1099/mgen.0.000338
https://www.biorxiv.org/content/10.1101/2024.04.29.591652v1.abstract

Response:

We thank the reviewer for mentioning these tools. Coinfinder and Goldfinder address a related but distinct problem: they detect statistically significant genome-wide gene co-occurrence patterns across a pangenome, relying on presence–absence matrices, without targeting any predefined genomic subset. In contrast, PANORAMA is designed to detect specific, architecturally defined multi-gene systems (such as defense systems) by leveraging gene adjacency in a pangenome graph, which enables recovery of complete system structures rather than pairwise co-occurrence signals. These tools are therefore complementary rather than competing: Coinfinder and Goldfinder are suited to exploratory, data-driven discovery of gene associations, whereas PANORAMA targets hypothesis-driven detection of known functional systems. We have added both tools in the Introduction to acknowledge this complementary landscape, alongside panModule, another tool exploiting gene co-localization in pangenomes:

> *Their strong genomic co-occurrence and co-localization make them amenable to systematic detection; tools such as Coinfinder, Goldfinder, and panModule exploit this property to identify conserved gene clusters across a set of genomes.*

Comment 4 - S1 Tab legend contains an error

Line 615, S1 legend - appears to be an error "Analy[s]es were performed on the , RAM disponible, type de machine"

Response:

We thank the reviewer for catching this error. The sentence was an incomplete placeholder left from an earlier draft. It has been corrected to provide the actual server specifications used for the analyses:

> **Revised S1 Tab caption (excerpt):** *Analyses were performed on two Intel Xeon Gold 6150 processors (36 cores), 376 GiB RAM, with Python 3.10.*

Comment 5 — Fig S2: labels not legible and interpretation not clear

Fig S2 labels are not legible and interpretation is not clear.

Response:

We thank the reviewer for this comment. Fig S2 is a static screenshot of an interactive figure automatically generated by PANORAMA using the Bokeh visualization library. Its purpose is to illustrate the format of PANORAMA's built-in output, not to convey a specific analytical result — detailed exploration is intended to be performed through the interactive version available in the Zenodo repository.

We acknowledge that this purpose was not sufficiently clear from the original caption. We have revised the caption to explicitly state that the figure illustrates PANORAMA's automated output format, to describe what rows, columns, and color intensity represent, and to direct readers to the interactive version for detailed exploration:

> **Revised S2 Fig caption:** ***Example of PANORAMA automated output: number of systems predicted per model across Enterobacteriaceae pangenomes.*** *This heatmap is automatically generated by PANORAMA using the Bokeh package. Each row corresponds to a species pangenome and each column to a defense system model; color intensity indicates the number of predicted systems (color scale, right). The figure is shown here to illustrate the format of PANORAMA's built-in visualization output. The full interactive version — enabling zooming, panning, and hovering to display exact values — is available in the Zenodo repository and can be opened directly in any standard web browser.*

Reviewer 2

Comment 1 — Pangenome definition is restricted to gene content

The pangenome is not only the whole gene set, but can be considered also as the whole genomic sequence set, including IS, regulatory regions, etc.

Response:

We thank the reviewer for this important clarification. We agree that the pangenome concept, in its broadest sense, encompasses all genomic sequences within a species or clade. The original sentence was too restrictive in implying a gene-only definition. We have revised the opening of the Introduction to present the full breadth of the pangenome concept first, and then introduce the core/accessory framework as a general way to describe genomic diversity across strains, without prematurely restricting to the gene level:

> **Revised text:** *The pangenome encompasses all genomic information within a species or clade, including coding regions, non-coding intergenic sequences, regulatory elements, and mobile genetic elements such as insertion sequences. It provides a holistic framework to*

capture and compare genomic diversity across strains, distinguishing a core component shared by all strains from a variable component found only in a subset of them, thereby deepening our understanding of microbial species evolution and adaptation.

Comment 2 — Pangenome graph type used by PANORAMA should be specified

The type of pangenome graph that PANORAMA deals with are gene adjacency graphs produced by PPanGGOLiN, but other types of pangenome graphs exist: k-mers, De Bruijn, vg… Authors should restrict their type of graph.

Response:

We thank the reviewer for this precise and important remark. We fully agree that "pangenome graph" is an umbrella term covering very different computational representations, and that the manuscript lacked the necessary distinction. We have added a sentence to the Introduction that explicitly contrasts sequence-level graph representations — such as De Bruijn graphs (e.g., Bifrost) and variation graphs (e.g., vg) — with gene-level adjacency graphs, and that positions PPanGGOLiN, and by extension PANORAMA, within the latter category. The description of PPanGGOLiN has also been updated to use the more precise term "gene adjacency graph":

> **Added sentence (Introduction, paragraph 2):** *Among the computational graph representations developed to analyze pangenomes, sequence-level graphs — such as De Bruijn graphs and variation graphs — encode genomic variation at the nucleotide level, while gene-level adjacency graphs represent gene family presence/absence and genomic contiguity across strains. Among the latter, PPanGGOLiN stands out…*

> **Updated PANORAMA description (Introduction, paragraph 4):** *…a powerful computational tool designed to harness bacterial gene-level pangenome graphs from large genomic datasets and enable comparisons across species to explore genomic diversity.…*

Comment 3 — Risk of using a representative sequence for HMM annotation

What are the risks (present all along the analyses) that using a representative sequence for HMM annotation? Is it possible that such a representative in a very large family lacks some specific subfunctions present in other copies?

Response:

We thank the reviewer for raising this important methodological point. Using a representative sequence is a deliberate default design choice that carries a theoretical risk: if a large gene family harbors functionally divergent subfamilies, the representative may not align well against HMMs targeting one of those subfunctions, leading to missed annotations. In practice, this risk is mitigated by the pangenome construction step: PPanGGOLiN builds gene families using default thresholds of 80% amino acid identity and 80% sequence coverage, which ensures a high degree of sequence similarity within each family and limits functional divergence between members.

Furthermore, PANORAMA provides two alternative annotation strategies. First, a consensus sequence derived from a multiple sequence alignment (MSA) of all family members can be used as the alignment target, better capturing the shared sequence space of the family. Second, HMM profiles can be aligned against all individual gene sequences within each family, ensuring that no divergent member is overlooked, at the cost of increased computational resources. In our testing across all species in this study, neither alternative substantially alters overall system detection outcomes compared to the default.

The Materials and Methods section has been updated to describe all three annotation strategies explicitly, and to note how the 80% identity/coverage threshold limits intra-family divergence:

> **Revised text (M&M — Functional annotation of pangenome gene families):** *By default, the protein sequence of each family's representative gene is aligned to the HMM profiles […] Since gene families in the pangenome are constructed with default thresholds of 80% amino acid identity and 80% sequence coverage, members within a family share a high degree of sequence similarity, which limits the risk that the representative sequence fails to capture the functional breadth of the family. Nevertheless, PANORAMA provides two additional alignment strategies […] First, a consensus sequence […] Second, HMM profiles can be aligned against all individual gene sequences within each family, ensuring that no divergent member is overlooked.*

Comment 4 — Annotation of novel genes absent from HMM databases

How do you deal with not-recorded HMM sequences? Let's say you have a very new type of bacteria, with very new functions — will the new genes be annotated?

Response:

This comment is closely related to Comment 3 and addresses the more general case of genes with no homologs in existing HMM databases. By design, PANORAMA — like all HMM-based annotation tools — cannot annotate gene families for which no profile exists. This is not solely a limitation: it provides an implicit validation guarantee, as detected systems are grounded in known, characterized protein families, reducing the risk of spurious predictions.

For use cases involving novel species or newly discovered functions, PANORAMA offers several complementary strategies. Users can bypass HMM annotation entirely by supplying gene family functional annotations directly as a tab-separated file, allowing integration of any external prediction source — including structure-based tools, protein language models, or custom databases. Furthermore, PANORAMA supports simultaneous annotation from multiple sources, so a standard database (e.g., DefenseFinder models) and a user-defined custom HMM library can be applied in a single run.

The Materials and Methods section has been updated to explicitly describe the external annotation bypass, the multi-source annotation capability, and the recommendation for custom databases when working with novel species or functions:

> **Revised text (M&M — Functional annotation of pangenome gene families):** *Gene family annotation can also be performed externally using alternative prediction methods, with results provided to PANORAMA as a tab-separated values (TSV) file. Multiple annotation sources can be used simultaneously, […] This is particularly relevant for functions not yet represented in existing HMM databases, for which external annotation tools or purpose-built HMM profiles can be used upstream of PANORAMA and supplied via this mechanism.*

Comment 5 - Include pangenome construction cost

For the benchmarking compared to Padloc and DefenseFinder, can you add also the CPU cost for computing the graph ? Your initial data is always the single genomes, not the graph, so it will be fairer to include the graph building in the comparison.

Response:

We thank the reviewer for this valid remark. We agree that, since the starting point for all tools is a set of raw genomes, a fully fair comparison should account for the complete pipeline, including PPanGGOLiN pangenome construction as a prerequisite for PANORAMA. We have therefore added a dedicated row for PPanGGOLiN (v2.3.0) in Table 2, reporting its runtime and peak memory usage on the 941 *P. aeruginosa* genomes.

We note, however, an important distinction that tempers this comparison: the PPanGGOLiN pangenome is built once per species and its output can be reused across any number of subsequent pangenome analyses — including different system models, updated databases, or comparative analyses. This amortizes its construction cost, whereas PADLOC and DefenseFinder must re-process all genomes from scratch for every new analysis. The table caption and the benchmark description in the Materials and Methods have been updated accordingly:

> **Revised M&M text:** *For completeness, the pangenome construction step performed by PPanGGOLiN is reported separately in S1 TAB as a prerequisite step for PANORAMA. Although raw genomes are indeed the starting point for all tools, we note that the PPanGGOLiN pangenome is built once per species and its output can be reused across any number of subsequent pangenome analyses, amortizing its construction cost.*

Comment 6 - Panorama cost of memory limit

Panorama is faster at the cost of more memory. Is there a limit for that memory use ?

Response:

Memory usage in PANORAMA is primarily driven by two factors: loading the pangenome content into memory, and the genomic context algorithm used to identify co-localized gene families. The computational cost of the latter scales with the `max_separation` parameter — which controls the maximum intergenic distance considered during context extraction — and with the number of families defined in the model. Models with a large number of families and a high transitivity setting will therefore require more memory. In practice, systems are defined by a restricted set of gene families in a limited genomic space; memory consumption is therefore not a limiting factor for their detection, even in large pangenomes

---

Comment 7 - Pangenome completeness imbalance biases cross-species comparison

The efficiency of Panorama is for me in the comparison of pangenomes. The method is very nice, however in the results themselves, the much higher number of E. coli (and thus the almost completion of the pangenome for it) will impair the comparison with much less numerous genomes, no ?

Response:

We thank the reviewer for this insightful observation. The concern is valid: a large pangenome such as that of *E. coli* (3,083 genomes) provides a more comprehensive representation of the accessory genome than less sampled species, which may bias cross-species comparisons. We acknowledge this as an inherent limitation of comparative pangenomics studies relying on publicly available genomes, where sampling depth is uneven across species. Genome dereplication to reduce redundancy within the *E. coli* dataset, or rarefaction analyses to assess how metrics change with pangenome size, would be relevant approaches to address this bias and are highlighted as perspectives for future work.

This findings warrants further investigation by conducting genome dereplication or rarefaction analysis to evaluate if this pattern remains robust in subsets of genomes.

Comment 8 - Intra-gene variation (SNPs/SVs) not captured at gene-family level

What about small SV and SNP within genes ? Can they be taken into account for evolutionary studies ?

Response:

By using gene families, small SV and SNP are diluted in the family and with the default parameters will certainly not be detected (this is linked to comments 3 and 4). However, by tuning the analyses (annotation of all genes, increasing identity and or coverage, using an external annotation) can potentially include them in the results and so be helpful in evolutionary studies.

Comment 9 — Justification of the CeCILL 2.1 license

Why using CeCILL 2.1 licence and not Apache or LGPL?

Response:

The choice of CeCILL 2.1 reflects two complementary considerations. First, PANORAMA was developed at CEA (Commissariat à l'Énergie Atomique), a French public research institution for which CeCILL — jointly created by CEA, CNRS, and INRIA — is the standard institutional open-source license, as it was specifically designed to comply with French law. Second, PANORAMA is built directly on PPanGGOLiN, which is also distributed under CeCILL; using the same license ensures full compatibility across the dependency chain and avoids any legal ambiguity. We note that CeCILL 2.1 is recognized by the Free Software Foundation as a free software license compatible with GNU GPL v2 and v3, and provides equivalent copyleft guarantees, ensuring that PANORAMA and any derivative works remain freely available to the community.

Comment 10 - Fig 9 legend contain an error

In the figure 9, there is no (4) part

Response:

We thank the reviewer for catching this inconsistency. The reference to step "(4)" in the Figure 9 legend was an outdated annotation from a previous version of the figure. It has been removed from the caption, which now correctly describes three sequential steps consistent with the figure content.

Comment 11 - Gene family fragmentation across divergent species due to clustering threshold

We thank the reviewer for raising this point. The gene family clustering methodology relies on pairwise sequence alignments between the representative sequences of each gene family across pangenomes. By default, PANORAMA applies a 50% identity threshold with 80% coverage. These parameters are consistent with those used in Uniclust50 (Mirdita *et al.*, 2017, *Nucleic Acids Research*, https://doi.org/10.1093/nar/gkw1081), a widely used protein sequence clustering database that demonstrates high functional annotation consistency at this threshold. For the study of moderately divergent species like those belonging to the same taxonomic family (e.g. Enterobacteriaceae), this choice is supported by the objective of grouping functionally equivalent protein homologs. For the analysis of more divergent species, lowering the identity threshold (e.g., to 30%) would be necessary; however, this would no longer ensure the reliable grouping of isofunctional homologs. To address this limitation, PANORAMA provides the option to incorporate external clustering results that preserve functional consistency, such as one based on KEGG Orthology (KO) families.
The optimal clustering strategy depends on the biological question and the level of species diversity to study. An evaluation of clustering methods is therefore beyond the scope of this manuscript, which focuses on the PANORAMA comparison framework. However, this represents a valuable direction for future work.

> **M&M — Gene families clustering**: *These thresholds are consistent with those used in Uniclust50, a widely used protein sequence clustering database that demonstrates high functional annotation consistency at 50\% identity.*
