# Supplementary material for: Panorama: A robust pangenome-based method for predicting and comparing biological systems across species
Source: PLoS Comput Biol. 2026 Jul 10;22(7):e1013856. doi: 10.1371/journal.pcbi.1013856 (PMC13379101; doi:10.1371/journal.pcbi.1013856)
Supplement: Peer review history

# Peer review: Decision Letter 0

**DOI:** 10.1371/journal.pcbi.1013856.r001

**Authors:** Ilya Ioshikhes, Sreekanth Thota

30 Mar 2026

PCOMPBIOL-D-25-02697

Panorama: a robust pangenome-based method for predicting and comparing biological systems across species

PLOS Computational Biology

Dear Dr. David Vallenet,

Thank you for submitting your manuscript to PLOS Computational Biology. After careful consideration, we feel that it has merit but does not fully meet PLOS Computational Biology's publication criteria as it currently stands. Therefore, we invite you to submit a revised version of the manuscript that addresses the points raised during the review process.

Please submit your revised manuscript by April 12th. If you will need more time than this to complete your revisions, please reply to this message or contact the journal office at ploscompbiol@plos.org. When you're ready to submit your revision, log on to https://www.editorialmanager.com/pcompbiol/ and select the 'Submissions Needing Revision' folder to locate your manuscript file.

Please include the following items when submitting your revised manuscript:

* A letter that responds to each point raised by the editor and reviewer(s). You should upload this letter as a separate file labeled 'Response to Reviewers'. This file does not need to include responses to formatting updates and technical items listed in the 'Journal Requirements' section below.

* A marked-up copy of your manuscript that highlights changes made to the original version. You should upload this as a separate file labeled 'Revised Manuscript with Track Changes'.

* An unmarked version of your revised paper without tracked changes. You should upload this as a separate file labeled 'Manuscript'.

If you would like to make changes to your financial disclosure, competing interests statement, or data availability statement, please make these updates within the submission form at the time of resubmission. Guidelines for resubmitting your figure files are available below the reviewer comments at the end of this letter.

We look forward to receiving your revised manuscript.

Kind regards,

Sreekanth Thota, Ph.D

Academic Editor

PLOS Computational Biology

Ilya Ioshikhes

Section Editor

PLOS Computational Biology

**Additional Editor Comments:**

Editor note:

1. Format Check and Revision:

Please ensure the entire manuscript is fully aligned with the PLOS Computational Biology scope and aims.

Please verify consistency of references and update formatting where necessary.

2. Figures and Figure Legends

Please conduct a thorough check of all figures and their legends to ensure there are no errors (e.g., incorrect labels, inconsistent numbering, or mismatched descriptions). Additionally, kindly provide the original high-resolution images to ensure print quality.

3. Language Polishing

This is the final language polishing before publication. Please refine the manuscript to ensure formal, accurate, and fluent English expression. You may consider seeking professional language editing services if necessary to eliminate grammatical errors, awkward phrasing, or ambiguity.

4. Author Information Verification

Please carefully confirm the accuracy of all author information, including full names, affiliations, email addresses, and ORCID. Ensure there are no typos or inconsistencies, as this information will be printed in the final publication.

**Journal Requirements:**

If the reviewer comments include a recommendation to cite specific previously published works, please review and evaluate these publications to determine whether they are relevant and should be cited. There is no requirement to cite these works unless the editor has indicated otherwise.

1) We ask that a manuscript source file is provided at Revision. Please upload your manuscript file as a .doc, .docx, .rtf or .tex. If you are providing a .tex file, please upload it under the item type u2018LaTeX Source Fileu2019 and leave your .pdf version as the item type u2018Manuscriptu2019.

2) We do not publish any copyright or trademark symbols that usually accompany proprietary names, eg ©,  ®, or TM  (e.g. next to drug or reagent names). Therefore please remove all instances of trademark/copyright symbols throughout the text, including:

- ® on pages: 22, and 23.

3) Please upload all main figures as separate Figure files in .tif or .eps format. For more information about how to convert and format your figure files please see our guidelines:

https://journals.plos.org/ploscompbiol/s/figures

4) We notice that your supplementary Figures, and Tables are included in the manuscript file. Please remove them and upload them with the file type 'Supporting Information'. Please ensure that each Supporting Information file has a legend listed in the manuscript after the references list.

5)  Please ensure that the funders and grant numbers match between the Financial Disclosure field and the Funding Information tab in your submission form. Note that the funders must be provided in the same order in both places as well.

6) Some material included in your submission may be copyrighted. According to PLOS's copyright policy, authors who use figures or other material (e.g., graphics, clipart, maps) from another author or copyright holder must demonstrate or obtain permission to publish this material under the Creative Commons Attribution 4.0 International (CC BY 4.0) License used by PLOS journals. Please closely review the details of PLOS's copyright requirements here: PLOS Licenses and Copyright. If you need to request permissions from a copyright holder, you may use the PLOS Content Copyright Permission Form Please respond directly to this email and provide any known details concerning your material's license terms and permissions required for reuse, even if you have not yet obtained copyright permissions or are unsure of your material's copyright compatibility. Once you have responded and addressed all other outstanding technical requirements, you may resubmit your manuscript within Editorial Manager. Potential Copyright Issues:

i) Figures 1, 9, and 10. Please confirm whether you drew the images / clip-art within the figure panels by hand. If you did not draw the images, please provide (a) a link to the source of the images or icons and their license / terms of use; or (b) written permission from the copyright holder to publish the images or icons under our CC BY 4.0 license. Alternatively, you may replace the images with open source alternatives. See these open source resources you may use to replace images / clip-art: - https://commons.wikimedia.org - https://openclipart.org/.

**Reviewers' comments:**

Reviewer's Responses to Questions

**Comments to the Authors:**

**Please note here if the review is uploaded as an attachment.**

Reviewer #1: Arnoux et al. present a cutting edge tool for functional annotation and comparison of bacterial pangenomes, incorporating detection of important gene clusters such as secretion systems, defense systems, and genes encoding key metabolic pathways - PANORAMA. The manuscript is a detailed and impressive study.

Discussion of how to create models would be useful. It is not clear how much of this is implemented within PANORAMA itself.

Other tools which may be relevant to cite include:

- Hamburger: https://www.nature.com/articles/s41467-022-32929-2 https://github.com/djw533/hamburger

- spacedust: https://www.nature.com/articles/s41592-025-02816-x https://github.com/soedinglab/spacedust

It may be relevant to note gene-gene co-occurrence detection methods such as Coinfinder or Goldfinder

https://www.microbiologyresearch.org/content/journal/mgen/10.1099/mgen.0.000338

https://www.biorxiv.org/content/10.1101/2024.04.29.591652v1.abstract

Line 615, S1 legend - appears to be an error “Analy[s]es were performed on the , RAM 615

disponible, type de machine”

Fig S2 labels are not legible and interpretation is not clear.

Reviewer #2: The manuscript by Arnoux et al deals with Panorama, a new and efficient method for comparing pangene adjacency graphs in bacteria. The paper is well written, methods clearly described and the examples used well organized and relevant. I have a very few comments on the paper and on the results, thus, recommend it for Minor Revisions

Introduction:

- The pangenome is not only the whole gene set, but can be considered also as the whole genomic sequence set, including IS, reg regions, etc...

- The type of pangenome graph that Panorama deals with are gene adjacency graphs produced by Ppanggolin, but other types of pangenome graphs exists: k-mers, DeBrujin, vg... Authors should restrict their type of graph.

Results

- What are the risks (present all along the analyses) that using a representative sequence for HMM annotation ? Is it possible that such representative in a very large family lacks some specific subfunctions present in other copies ?

- How do you deal with not-recorded in HMM sequences ? Let's say you have a very new type of bacteria, with very new functions, will the new genes be annotated ?

- For the benchmarking compared to Padloc and DefenseFinder, can you add also the CPU cost for computing the graph ? Your initial data is always the single genomes, not the graph, so it will be fairer to include the graph building in the comparison.

-Panorama is faster at the cost of more memory. is there a limit for that memory use ?

- The efficiency of Panorama is for me in the comparison of pangenomes. The method is very nice, however in the results themselves, the much higher number of E. coli (and thus the almost completion of the pangenome for it) will impair the comparison with much less numerous genomes, no ?

Discussion

- What about small SV and SNP within genes ? Can they be taken into account for evolutionary studies ?

M&M

-Why using Cecill2.1 licence and not Apache or LGPL ?

- In the figure 9, there is no (4) part

- Page 20, when clustering the gene families, it is possible that two clusters from the same family did not recognize between two highly divergent species. Can you test it ?

**********

**Have the authors made all data and (if applicable) computational code underlying the findings in their manuscript fully available?**

The PLOS Data policy requires authors to make all data and code underlying the findings described in their manuscript fully available without restriction, with rare exception (please refer to the Data Availability Statement in the manuscript PDF file). The data and code should be provided as part of the manuscript or its supporting information, or deposited to a public repository. For example, in addition to summary statistics, the data points behind means, medians and variance measures should be available. If there are restrictions on publicly sharing data or code —e.g. participant privacy or use of data from a third party—those must be specified.

Reviewer #1: Yes

Reviewer #2: Yes

**********

PLOS authors have the option to publish the peer review history of their article (what does this mean?). If published, this will include your full peer review and any attached files.

If you choose “no”, your identity will remain anonymous but your review may still be made public.

**Do you want your identity to be public for this peer review?**  For information about this choice, including consent withdrawal, please see our Privacy Policy.

Reviewer #1: **Yes:**  Zachary Ardern

Reviewer #2: **Yes:**  Francois Sabot

**Figure resubmission:**

-->While revising your submission, we strongly recommend that you use PLOS’s NAAS tool (https://ngplosjournals.pagemajik.ai/artanalysis) to test your figure files. NAAS can convert your figure files to the TIFF file type and meet basic requirements (such as print size, resolution), or provide you with a report on issues that do not meet our requirements and that NAAS cannot fix.

After uploading your figures to PLOS’s NAAS tool - https://ngplosjournals.pagemajik.ai/artanalysis, NAAS will process the files provided and display the results in the "Uploaded Files" section of the page as the processing is complete. If the uploaded figures meet our requirements (or NAAS is able to fix the files to meet our requirements), the figure will be marked as "fixed" above. If NAAS is unable to fix the files, a red "failed" label will appear above. When NAAS has confirmed that the figure files meet our requirements, please download the file via the download option, and include these NAAS processed figure files when submitting your revised manuscript.

**Reproducibility:**

To enhance the reproducibility of your results, we recommend that authors of applicable studies deposit laboratory protocols in protocols.io, where a protocol can be assigned its own identifier (DOI) such that it can be cited independently in the future. Additionally, PLOS ONE offers an option to publish peer-reviewed clinical study protocols. Read more information on sharing protocols at https://plos.org/protocols?utm_medium=editorial-email&utm_source=authorletters&utm_campaign=protocols

# Author comment: Author response to Decision Letter 1

**DOI:** 10.1371/journal.pcbi.1013856.r002

1 Jun 2026

Attachment Submitted filename: Response2Reviewers.pdf

# Editor report: Decision Letter 1

**DOI:** 10.1371/journal.pcbi.1013856.r003

**Authors:** Ilya Ioshikhes, Sreekanth Thota

21 Jun 2026

Dear David Vallenet,

We are pleased to inform you that your manuscript 'Panorama: a robust pangenome-based method for predicting and comparing biological systems across species' has been provisionally accepted for publication in PLOS Computational Biology.

Before your manuscript can be formally accepted you will need to complete some formatting changes, which you will receive in a follow up email. A member of our team will be in touch with a set of requests.

Please note that your manuscript will not be scheduled for publication until you have made the required changes, so a swift response is appreciated.

IMPORTANT: The editorial review process is now complete. PLOS will only permit corrections to spelling, formatting or significant scientific errors from this point onwards. Requests for major changes, or any which affect the scientific understanding of your work, will cause delays to the publication date of your manuscript.

Should you, your institution's press office or the journal office choose to press release your paper, you will automatically be opted out of early publication. We ask that you notify us now if you or your institution is planning to press release the article. All press must be co-ordinated with PLOS.

Thank you again for supporting Open Access publishing; we are looking forward to publishing your work in PLOS Computational Biology.

Best regards,

Sreekanth Thota, Ph.D

Academic Editor

PLOS Computational Biology

Ilya Ioshikhes

Section Editor

PLOS Computational Biology

***********************************************************

Dear Dr David Vallenet,

I am pleased to inform you that your manuscript has been accepted for publication in the current form.

# Editor report: Acceptance letter

**DOI:** 10.1371/journal.pcbi.1013856.r004

**Authors:** Ilya Ioshikhes, Sreekanth Thota

PCOMPBIOL-D-25-02697R1

Panorama: a robust pangenome-based method for predicting and comparing biological systems across species

Dear Dr Vallenet,

I am pleased to inform you that your manuscript has been formally accepted for publication in PLOS Computational Biology. Your manuscript is now with our production department and you will be notified of the publication date in due course.

The corresponding author will soon be receiving a typeset proof for review, to ensure errors have not been introduced during production. Please review the PDF proof of your manuscript carefully, as this is the last chance to correct any errors. Please note that major changes, or those which affect the scientific understanding of the work, will likely cause delays to the publication date of your manuscript.

Soon after your final files are uploaded, unless you have opted out, the early version of your manuscript will be published online. The date of the early version will be your article's publication date. The final article will be published to the same URL, and all versions of the paper will be accessible to readers.

For Research, Software, and Methods articles, you will receive an invoice from PLOS for your publication fee after your manuscript has reached the completed accept phase. If you receive an email requesting payment before acceptance or for any other service, this may be a phishing scheme. Learn how to identify phishing emails and protect your accounts at https://explore.plos.org/phishing.

Thank you again for supporting PLOS Computational Biology and open-access publishing. We are looking forward to publishing your work!

With kind regards,

Sharmila Kamatchi

PLOS Computational Biology | Carlyle House, Carlyle Road, Cambridge CB4 3DN | United Kingdom ploscompbiol@plos.org | Phone +44 (0) 1223-442824 | ploscompbiol.org | @PLOSCompBiol
